# Comparative and functional genomics of the protozoan parasite *Babesia divergens* highlighting the invasion and egress processes

**DOI:** 10.1371/journal.pntd.0007680

**Published:** 2019-08-19

**Authors:** Luis Miguel González, Karel Estrada, Ricardo Grande, Verónica Jiménez-Jacinto, Leticia Vega-Alvarado, Elena Sevilla, Jorge de la Barrera, Isabel Cuesta, Ángel Zaballos, José Manuel Bautista, Cheryl A. Lobo, Alejandro Sánchez-Flores, Estrella Montero

**Affiliations:** 1 Laboratorio de Referencia e Investigación en Parasitología, Centro Nacional de Microbiología, ISCIII Majadahonda, Madrid, Spain; 2 Unidad Universitaria de Secuenciación Masiva y Bioinformática, Instituto de Biotecnología, Cuernavaca, México; 3 Instituto de Ciencias Aplicadas y Tecnología, UNAM, Ciudad de México, México; 4 Unidad de Bioinformática, Área de Unidades Centrales Científico-Técnicas, ISCIII, Majadahonda, Madrid, Spain; 5 Unidad de Genómica, Área de Unidades Centrales Científico-Técnicas, ISCIII, Majadahonda, Madrid, Spain; 6 Department of Biochemistry and Molecular Biology & Research Institute Hospital 12 de Octubre, Facultad de Veterinaria, Universidad Complutense de Madrid, Madrid, Spain; 7 Blood Borne Parasites, LFKRI, New York Blood Center, New York, New York, United States of America; University of Agricultural Sciences and Veterinary Medicine Cluj-Napoca, Life Science Institute, ROMANIA

## Abstract

Babesiosis is considered an emerging disease because its incidence has significantly increased in the last 30 years, providing evidence of the expanding range of this rare but potentially life-threatening zoonotic disease. *Babesia divergens* is a causative agent of babesiosis in humans and cattle in Europe. The recently sequenced genome of *B*. *divergens* revealed over 3,741 protein coding-genes and the 10.7-Mb high-quality draft become the first reference tool to study the genome structure of *B*. *divergens*. Now, by exploiting this sequence data and using new computational tools and assembly strategies, we have significantly improved the quality of the *B*. *divergens* genome. The new assembly shows better continuity and has a higher correspondence to *B*. *bovis* chromosomes. Moreover, we present a differential expression analysis using RNA sequencing of the two different stages of the asexual lifecycle of *B*. *divergens*: the free merozoite capable of invading erythrocytes and the intraerythrocytic parasite stage that remains within the erythrocyte until egress. Comparison of mRNA levels of both stages identified 1,441 differentially expressed genes. From these, around half were upregulated and the other half downregulated in the intraerythrocytic stage. Orthogonal validation by real-time quantitative reverse transcription PCR confirmed the differential expression. A moderately increased expression level of genes, putatively involved in the invasion and egress processes, were revealed in the intraerythrocytic stage compared with the free merozoite. On the basis of these results and in the absence of molecular models of invasion and egress for *B*. *divergens*, we have proposed the identified genes as putative molecular players in the invasion and egress processes. Our results contribute to an understanding of key parasitic strategies and pathogenesis and could be a valuable genomic resource to exploit for the design of diagnostic methods, drugs and vaccines to improve the control of babesiosis.

## Author summary

Babesiosis has long been recognized as an economically important disease of cattle, but only in the last 40 years has *Babesia* been recognized as an important pathogen in humans. Babesiosis in humans is caused by one of several species (*B*. *microti*, *B*. *divergens*, *B*. *dun*cani and *B*. *venatorum*). The complete *Babesia* lifecycle requires two hosts, the ixodid ticks and a vertebrate host. It is the parasite's ability to first recognize and then invade host erythrocytes that is central to the pathogenesis of babesiosis. Once inside the cell, the parasite begins a cycle of maturation and growth, resulting in merozoites that egress from the red blood cells (RBCs) and seek new, uninfected RBCs to invade, perpetuating the infection. To better understand this asexual lifecycle, the authors focused on the parasite genome and transcriptome of the asexual erythrocytic forms of *B*. *divergens*. Through this functional and comparative genomic approach, the authors have identified genes putatively involved in invasion, gliding motility, moving junction formation and egress, providing new insights into the molecular mechanisms of these processes necessary for *B*. *divergens* to survive and propagate during its life cycle.

## Introduction

Babesiosis is a worldwide emerging infectious disease [1] caused by a protozoan parasite of the genus *Babesia* which is naturally transmitted by ixodid ticks and infects vertebrate erythrocytes. Parasite infection of natural hosts such as in cattle causes bovine babesiosis, resulting in high economic losses. Recently, the severity of human infection has also become apparent [2]. The four identified *Babesia* species confirmed to infect humans to date are *B*. *microti*, *B*. *divergens*, *B*. *duncani*, and *B*. *venatorum* [2,3]. *B*. *microti* has a worldwide distribution while *B*. *divergens* is a causative agent of babesiosis in humans and cattle in Europe [3].

Symptoms range from asymptomatic to flu-like symptoms in healthy individuals. However, in asplenic, immune-compromised patients, children, elderly people and patient who are experiencing *B*. *divergens* infection, the disease can be severe. Thus, human babesiosis results in hemolytic anemia, jaundice and hemoglobinuria due to the lytic effects of parasite multiplication in the red blood cells (RBC). *B*. *divergens* infections are therefore considered medical emergencies and patients require immediate treatment [3].

As evident from its status as an emerging disease, the number of human babesiosis cases has experienced a global increase [1]. This number may still not fully reflect the actual numbers of infections as many cases go unreported or are misdiagnosed because the non-specific symptoms of babesiosis can hinder accurate diagnosis. European cases in particular may reflect this situation because babesiosis is not a notifiable disease in the continent and several cases likely go unreported.

This is supported by seroprevalence studies that indicate that the percentage of the European population infected with *Babesia* is much higher than the low clinical incidence and case reports to date [4]. Notably, these anti-babesial antibody studies also indicate that the detected antibodies were acquired by people who were exposed to the *Babesia* parasites [4]. In fact, antibody testing is important in identifying asymptomatic and mild cases when parasites may not be detectable in a blood smear or by PCR, suggesting the need for active surveillance programs to accurately detect babesiosis in Europe.

Originally endemic in just a few regions on the world, including Europe [3,4] and EEUU [2] the steady increase of cases in other areas of the world (Asia, Africa, Australia, Canada, Egypt and South America) indicates its zoonotic potential. Notably, the presence of *B*. *divergens* outside Europe and North Africa was recently confirmed in China and Japan [5,6].

Onset of symptoms in the host results from the continuous invasion and destruction of RBCs by the parasite. The invasion of RBCs by the *B*. *divergens* free merozoite is an active process [7] that exploits an original locomotion mechanism called gliding motility, an intimate tight connection between the parasite and the erythrocyte known as a moving junction and a coordinated secretion of proteins from apical organelles (rhoptries, micronemes, dense granules or spherical bodies according to the species) [8–10]. Once hidden within the host cell, the intraerythrocytic lifestyle contributes to protect parasites from the interactions of anti-babesial antibodies capable of recognized and block the antigens of the free merozoite surface. Parasites gain nutrients and multiply by binary fission resulting in a considerably complex pleomorphic process that yields different intraerythrocytic parasite stages leading to the development of merozoites that exit to invade new RBCs and thus propagate [7,11].

*In vitro*, this proliferative cycle [12] has been studied to investigate biological processes such as invasion, multiplication and egress [7,12–14]. However, the dynamics and regulation of these processes are still largely unexplored at the molecular level in *B*. *divergens*.

To gain new insight into this emerging but neglected pathogen, the genome of *B*. *divergens* human isolate (Bd 87 strain) was recently sequenced and assembled by our group using reads produced by three next-generation sequencing (NGS) platforms. The draft genome (publicly available under accession number CCSG01000001 to CCSG01000514) was approximately 10.7-Mb in 514 scaffolds and revealed over 3,741 protein coding-genes [15].

In this study, we significantly improve the quality of assembly of the *B*. *divergens* genome by exploiting our previous sequence data using new computational tools and assembly strategies. We also report transcriptional data from two different stages of the *B*. *divergens in vitro* asexual lifecycle: i) free extra-cellular merozoite and ii) intraerythrocytic stage. Using genomic and transcriptomic data we provide an annotation-directed improved genome sequence assembly [16] which enable us to explore the synteny between *B*. *divergens* and other *Babesia* species. Moreover, this functional and comparative genomics approach alongside the gene expression profile of the two stages, highlight molecular features associated with key processes to the survival of the parasite, including invasion, gliding motility, moving junction formation and egress.

## Results and discussion

### Annotation-directed improvement of *Babesia divergens* genome

Previously, we reported a draft genome [15] for *B*. *divergens*, assembled using a hybrid approach with three different platforms and sequencing depths: Illumina (~310x); 454 (~20x); Pacific Biosciences (PacBio) (~20x). Reassembly of the genome delivered larger contigs and scaffolds with chromosome lengths. As mentioned, the assembly continuity has been improved here, as shown in Table 1, where the number of contigs and scaffolds has been reduced and their average length has increased. The latest version of the genome can be found under the accession number GCA001077455 (CCSG02000001-CCSG02000141).

**Table 1:** Assembly and parameters for *B*. *divergens* genome.

	New version	Previous version
Total bases in the assembly (Mb)	**9.73**	10.7
No. of scaffolds	**141**	514
Scaffold N50 (Mb) / L50	1.08 / 4	1.08 / 4
Average length (kb)	**68.97**	21.01
Largest scaffold (Mb)	2.20	2.20
GC% content	**43**	45.3
No. of contigs	**350**	709
Contig N50 (kb) / L50	144.47 / 19	177.92 / 18
Average length (bp)	**21,790**	14,726
Largest contig (bp)	**560,835**	551,113
**Predicted gene models of the new version**		
Total gene models / annotated	4,546 / 3,386	
Exon mean / median / mode (bp)	727 / 352 / 65	
Intron mean / median / mode (bp)	328 / 105 / 36	
Average exon per gene	1.68	

The differences between the new and previous assembly are marked in bold.

The new assembly showed a better continuity that can be inferred from the reduced number of scaffolds and contigs and their higher average length. The total bases in the assembly was reduced since several redundant contigs that were generated in the previous assembly, were accurately resolved, although the contig N50 value decreased which is attributable to a more uniform length distribution and probably because some contigs were broken in misassembled regions.

### Comparative genomics

In the previous assembly, we generated 5,000 gene models, but in this work, the gene prediction was improved by using RNA sequencing (RNAseq) data from both free merozoites and intraerythrocytic parasite stages (details are explained below) and the corroboration of predicted genes by comparing their products against proteins from 17 different *Babesia* species. From a total of 40 million paired-end reads, 90% of these reads were successfully mapped to the genome, which allowed the validation of the 4,546 gene models. Additionally, genes were corroborated by ensuring that they had a protein ortholog with at least other *Babesia* species (S1 Table).

Interestingly, from all the 17 *Babesia* species analyzed in the protein orthologous comparison, *B*. *microti* and *B*. *bovis* had the most orthologous in common with *B*. *divergens* (3,251 and 3,738, respectively). Taking in account this orthology information, we could support the annotation of 3,386 proteins in *B*. *divergens* which we considered as high confidence annotation.

We also analyzed the clustering of orthologous proteins between the three species and found 2,660 groups where 1,427 were the core genes in the three species (S1 Table). According to our analysis, *B*. *bovis* presented 18 clusters with gene duplications while *B*. *microti* and *B*. *divergens* presented only 7 clusters with duplicated genes.

**Fig 1:**
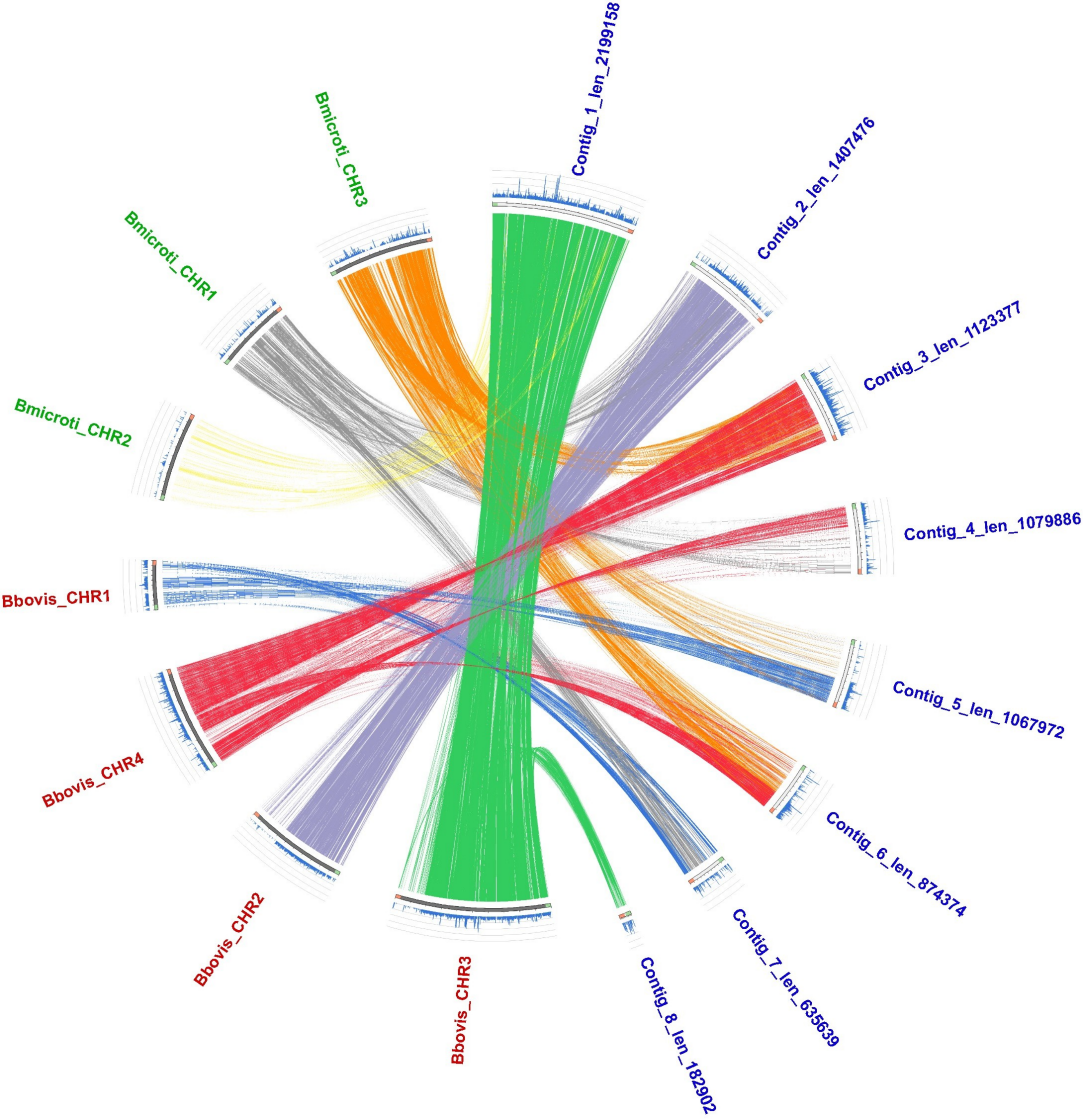
Circos plot of *B*. *divergens* synteny with *B*. *bovis* and *B*. *microti* chromosomes. Lines show the one-to-one ortholog comparison from each contig to chromosomes in other species. *B*. *divergens* contig names are indicated in blue, *B*. *bovis* chromosome names in red and *B*. *microti* chromosome names in green.

As a result of the ortholog comparison from the gene model prediction improvement, we decided to compare our latest assembly against the genomes of *B*. *bovis* and *B*. *microti* to explore the synteny between organisms. In Fig 1, the one-to-one orthologous connecting the chromosomes from *B*. *divergens* to *B*. *bovis* and *B*. *microti* are depicted, allowing us to observe that some of our scaffolds have similar chromosome resolution reported for the other species [17,18]. Interestingly, the arrangements in *B*. *divergens* are more similar to the organization of *B*. *bovis* than *B*. *microti*, especially between chromosomes 2 and 3 of the former species. Contigs 1 and 2 (~2.2 and 1.4 Mb) of our improved assembly have a high correspondence to *B*. *bovis* chromosomes 3 and 2, respectively. Contig 3 (1.12 Mb) has correspondence to the largest contig of chromosome 4, where we can observe a possible inversion and some regions rearranged in Contigs 4 and 6 (1.07 and 0.87 Mb). Notably, we observed some correspondence to chromosomes 1 and 3 from *B*. *microti*, with Contigs 3, 4, 5 (1.06 Mb), 6 and 7 (0.63 Mb), suggesting significant rearrangements between the two species. Chromosome 2 from *B*. *microti* presented some correspondence to Contig 1, but not as much as observed with *B*. *bovis* chromosome 3. It can be inferred that *B*. *microti* has more genomic rearrangements in comparison to *B*. *bovis* and *B*. *divergens*, which presented a higher number of syntenic regions. In fact, previous phylogenetic investigations confirmed that *B*. *microti* constitutes a piroplasmid lineage distinct from *B*. *bovis* and *B*. *divegens* [19]. It is noteworthy that the three genomes were sequenced and assembled using different sequencing technologies and assembly algorithms, which could hinder the synteny comparison. Since none of them have a finished genome quality, there will be limitations due to missing parts in each genome.

Additionally, we performed a pulse field gel electrophoresis (PFGE) for the genomic DNA and the results showed 4 probable chromosomes with the following approximate sizes of 1.05 (chromosome 1), ~1.7–1.8 (chromosome 2), ~2.9–3.0 (chromosome 3) and 4 Mb (chromosome 4) (S1 Fig). The size and number of chromosomes are also similar to those reported for the *B*. *bovis* genome [20] where chromosomes 1, 2, 3 and 4 are estimated to be 1.4, 2.0, 2.8 and 3.2 million base pairs, respectively. These results suggest that the probable ordering for our assembly, where we could assign contigs/scaffolds into chromosomes, is very similar to that observed in *B*. *bovis*. It is important to mention that although the *B*. *bovis* genome is the best *Babesia* reference genome available, only chromosomes 2 and 3 are finished and the rest are reported in several contigs [17]. In these cases, the use of genetic markers or FISH techniques [21] are necessary to corroborate the correspondence of the assembly into chromosomes and have achieve better synteny resolution.

### Sequencing and dynamic transcriptome analysis of *B*. *divergens* asexual cycle

**Fig 2:**
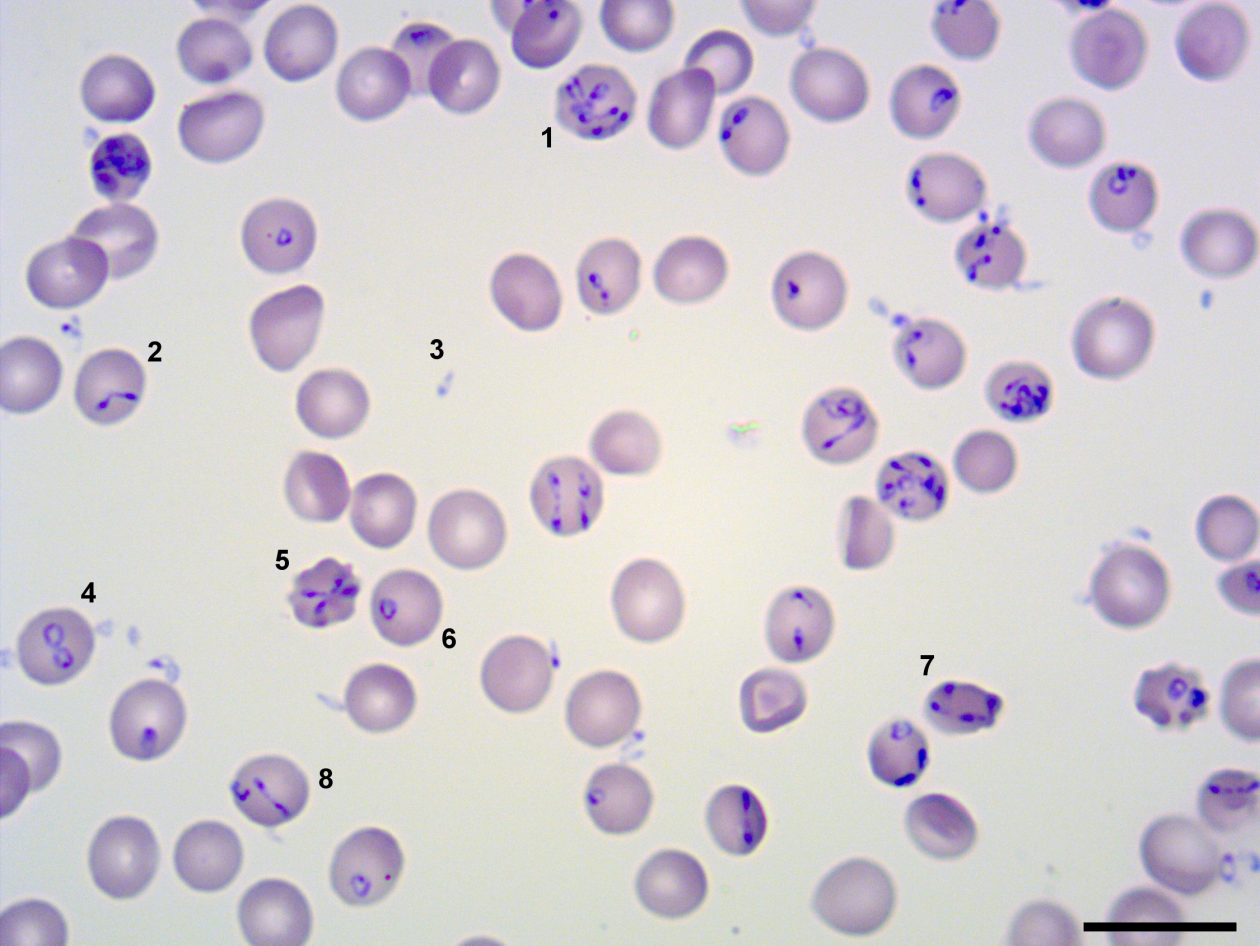
*B*. *divergens* free merozoites and intraerythrocytic stages observed in *in vitro* cultures at ≈ 40% of parasitemia by Giemsa staining. Figure shows free merozoites and intraerythrocytic parasite stages: (1) Multiple parasites stage formed by more than four parasites within the RBC, (2) paired pyriform, (3) free merozoite, (4) double trophozoites, (5) tetrad (6) single trophozoite, (7) double paired pyriforms, (8) transient morphological combination. Bar 15 μm.

Free merozoites and intraerythrocytic parasites (Fig 2) represent the result of the cyclic processes of invasion and egress that *B*. *divergens* undergoes during its asynchronous asexual life cycle. Hence, we have sequenced the transcriptomes of both free extracellular parasites and intraerythrocytic stages using Illumina sequencing (S2 and S3 Tables).

To profile both transcriptomes, we isolated mRNA from two biological replicates to generate two independent RNAseq libraries for each stage.

We compared the RNAseq expression profile of both free merozoite and intraerythrocytic parasite stages using three different algorithms: DEseq, EdgeR and NOIseq. The NOIseq analysis yielded 1,441 differentially expressed genes, 734 of them were up-regulated and 707 genes down-regulated in the intraerythrocytic parasite stage (S4–S6 Tables).

Based on these data and considering that molecular studies in term of host cell invasion and egress are limited in *Babesia* [22], we restricted our results to 45 genes of interest putatively related to both processes. These genes were manually inspected and curated using Artemis. Of note, 33 of the genes were confirmed by at least one of the three different algorithms and their expression levels were validated for free merozoite and intraerythrocytic stages using real-time quantitative reverse transcription PCR (qRT-PCR) (Table 2).

**Table 2:** List of genes exhibiting differential expression between *B*. *divergens* intraerythrocytic parasites and free merozoites.

**Up-regulated genes in the intraerythrocytic stage**			
**INVASION**			
**Gene ID**	**Annotation**	**RNAseq prediction** **|1|2|3|**	**Real-time** **qRT-PCR** **p-value**
BDIVROU_0113100.t1.2	rhoptry-associated protein 1b, RAP-1b	|-|-|+|	<0.0001
BDIVROU_0126000.t1.2	apical membrane antigen 1, BdAMA1	|-|-|+|	0.0047
BDIVROU_0272901.t1.2	gliding associated protein 45, GAP45	|-|-|+|	0.0114
BDIVROU_0259100.t1.2	rhoptry neck protein 2, RON2	|+|+|+|	0.0006
BDIVROU_0142300.t1.2	rhoptry neck protein 5, RON5	|-|-|+|	0.0035
BDIVROU_0113100.t1.2	rhoptry-associated protein 1, RAP-1	|-|-|+|	<0.0001
BDIVROU_0156200.t1.2	thrombospondin-related apical membrane protein, TRAP	|-|-|+|	0.0071
BDIVROU_0191501.t1.2	rhoptry neck protein 4, RON4	|-|-|+|	0.0013
BDIVROU_0182900.t1.2	37-kDa glycosylphosphatidylinositol-anchored surface protein, Bd37	|+|-|+|	0.0017
BDIVROU_0025700.t1.2	Actin	|-|-|+|	0.0005
BDIVROU_0052401.t1.2	gliding associated protein 40, GAP40	|-|-|+|	0.0014
BDIVROU_0412101.t1.2	myosin light chain1, MLC1	|-|-|+|	0.0128
BDIVROU_0073701.t1.2	F-actin–binding protein or coronin	|-|-|+|	0.0128
BDIVROU_0133800.t3.2	myosin chain B, MYOB	|-|-|+|	0.0198
BDIVROU_0130101.t1.2	calcium-dependent protein kinase 4, CDPK-4	|-|-|+|	0.0053
BDIVROU_0124800.t1.2	phosphoinositide phospholipase C, PI-PLC	|-|-|+|	0.0384
BDIVROU_0096510.t1.2	actin-depolymerizing factor, ADF or cofilin	|-|-|+|	0.0019
BDIVROU_0339200.t1.2	gliding associated protein, GAP50	|-|-|+|	<0.0001
BDIVROU_0339410.t1.2	protein kinase G, PKG	|-|-|+|	0.0189
BDIVROU_0242901.t1.2	myosin A, MYOA	|-|-|+|	<0.0001
BDIVROU_0224310.t1.2	Ser/Thr phosphatase, PP1 or calcineurin	|-|-|+|	0.0002
BDIVROU_0279300.t1.2	profilin	|-|-|+|	0.0014
BDIVROU_0152200.t1.2	glideosome-associated connector, GAC	|+|+|+|	<0.0001
BDIVROU_0183000.t1.2	50-kDa surface protein, BdP50	|-|-|+|	0.0117
BDIVROU_0165301.t1.2	spherical body protein 3, SPB3	|-|-|+|	0.0167
BDIVROU_0117600.t1.2	RAP-1 related antigen, RRA	|-|-|+|	0.023
**EGRESS**			
BDIVROU_0173601.t1.2	mac/perforin protein 4, MAC4	|-|-|+|	<0.0001
BDIVROU_0065210.t1.2	mac/perforin protein 3, MAC3	|-|-|+|	<0.0001
BDIVROU_0308600.t1.1	papain-2	|-|-|+|	0.0069
**Down-regulated genes in the intraerythrocytic stage**			
**INVASION**			
**Gene ID**	**Annotation**	**RNAseq prediction** **|1|2|3**|	**Real-time** **qRT-PCR** **p-value**
BDIVROU_0278701.t1.2	rhomboid-like protease 4.1, ROM4.1	|+|-|+|	0.0008
BDIVROU_0230400.t1.2	rhomboid-like protease 4.4, ROM4.4	|+|-|+|	0.001
BDIVROU_0280000.t1.2	subtilisin-like serine protease, BdSUB1	|-|-|+|	<0.0001
**EGRESS**			
BDIVROU_0161201.t1.2	mac/perforin protein 2, MAC2	|-|+|+|	0,0076

Differentially expressed genes were validated by RNAseq and confirmed by three different algorithms using a statistical cutoff-line for each one: 1 = DESeq padj < = 0.05; 2 = EdgeR, FDR < = 0.05, 3 = NOISeq, Prob > = 0.9. To verify RNA-seq results, real-time qRT-PCR was conducted for all of the genes. The +/- marks indicate when a method detected or not a gene as differential expressed according to their cutoff-line.

**Fig 3:**
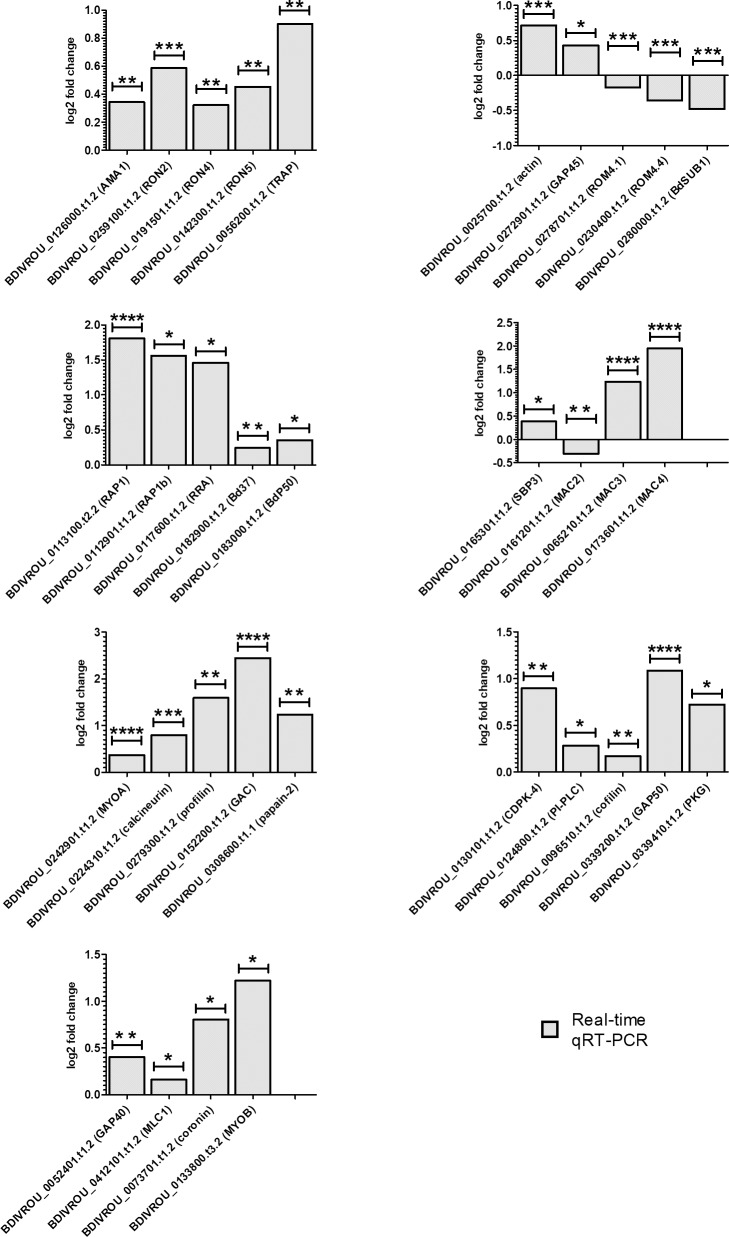
Transcriptomic profiling of both intraerythrocytic stages (above) and free merozoite (below) validated by RNAseq and real-time qRT-PCR. The values above the X-axis indicate the intraerythrocytic parasite up regulated genes and the values below the X-axis indicate free merozoite up regulated genes. Differential expression profile in both stages is denoted with 1, 2, 3 or 4 asterisks.

The real-time qRT-PCR analysis revealed that 26 genes putatively related to the components of the molecular invasion machinery and 3 genes related to the egress process (Table 2) [22–25] were upregulated between 0.2 and 2-fold in the intraerythrocytic stage. This result showed a moderately increased expression level of these genes in the intraerythrocytic stage compared with the free merozoite stage (Table 2 and Fig 3). However, 4 genes encoding proteases putatively involved in invasion or egress: i) BDIVROU_0278701.t1.2 (rhomboid-like protease 4.1, ROM4.1), ii) BDIVROU_0230400.t1.2 (rhomboid-like protease 4.4, ROM4.4), iii) BDIVROU_0280000.t1.2 (subtilisin-like serine protease, BdSUB1) and iv) BDIVROU_0161200.t1.1 (mac/perforin protein 2, MAC2) [26–29] were modestly induced between 0.2 and 0.5-fold in the free merozoite (Table 2 and Fig 3).

RNASeq and real-time qRT-PCR findings indicated that genes of specialized *B*. *divergens* functions could be present in both the free merozoite and intraerythrocytic stages, suggesting that the majority of the genome is utilized throughout the complete asexual lifecycle of this parasite. Notably, the upregulation of some genes in the intraerythrocytic stage could imply a moderately increased need for invasion, gliding motility, and egress protein expression, before these processes occur (Table 2). Alternatively, a moderate or less marked induction of the same genes in the free merozoite would be required to maintain baseline expression level during the processes. Moreover, this expression profile suggests that the intraerythrocytic parasite could harbor proteins in its apical organelles that are required during the rapid processes of invasion and egress, thus ensuring that the complex and specialized mechanisms of invasion, gliding, and egress are primed and function successfully at the appropriate time. Surprisingly, the free merozoites also exhibit qualitatively a similar expression profile, although the transcripts are at lower levels. This may be linked to the short lifecycle of the parasite and may reflect that these transcripts need to be present to accommodate the requirement for the various proteins as soon as the parasite invades. It will be of interest to check if the protein levels are also equivalent between these two parasite stages.

On the other hand, since the asexual life cycle of *B*. *divergens* is asynchronous, there are simultaneously intraerythrocytic parasites at different stages of their development, ranging from parasites which have just invaded new erythrocytes to parasites which are dividing or egressing from the host cell. As a result, there are not apparent abrupt changes in the expression profile between intracellular and extracellular stages. This is reflected in the data dispersion observed for free merozoites replicates in the principal component analysis (PCA) and multidimensional scaling (MDS) plots (S2 Fig.) generated by DESeq2 and EdgeR, respectively. However, for those proteins transcribed in two different phases of the lifecycle, i.e. when the parasite is outside the RBC and when it is inside the RBC, regardless their expression level, the research could lead towards the development of vaccines and new treatments capable of targeting several different stages at the same time, as previously suggested for *P*. *falciparum* [30].

### The composition and assembly of the invasion and egress molecular machineries of *B*. *divergens*

Finally, on the basis of this genomic approach and in the absence of models of invasion and egress available for *B*. *divergens*, we have proposed the following potential molecular models according to the *B*. *divergens* genes identified and validated in this work (S7 and S8 Tables) and following the general prototypes suggested for the apicomplexan parasites *Plasmodium* and *Toxoplasma* [22–25].

a) *B. divergens* genes associated with calcium (Ca^2+^) in invasion

**Fig 4:**
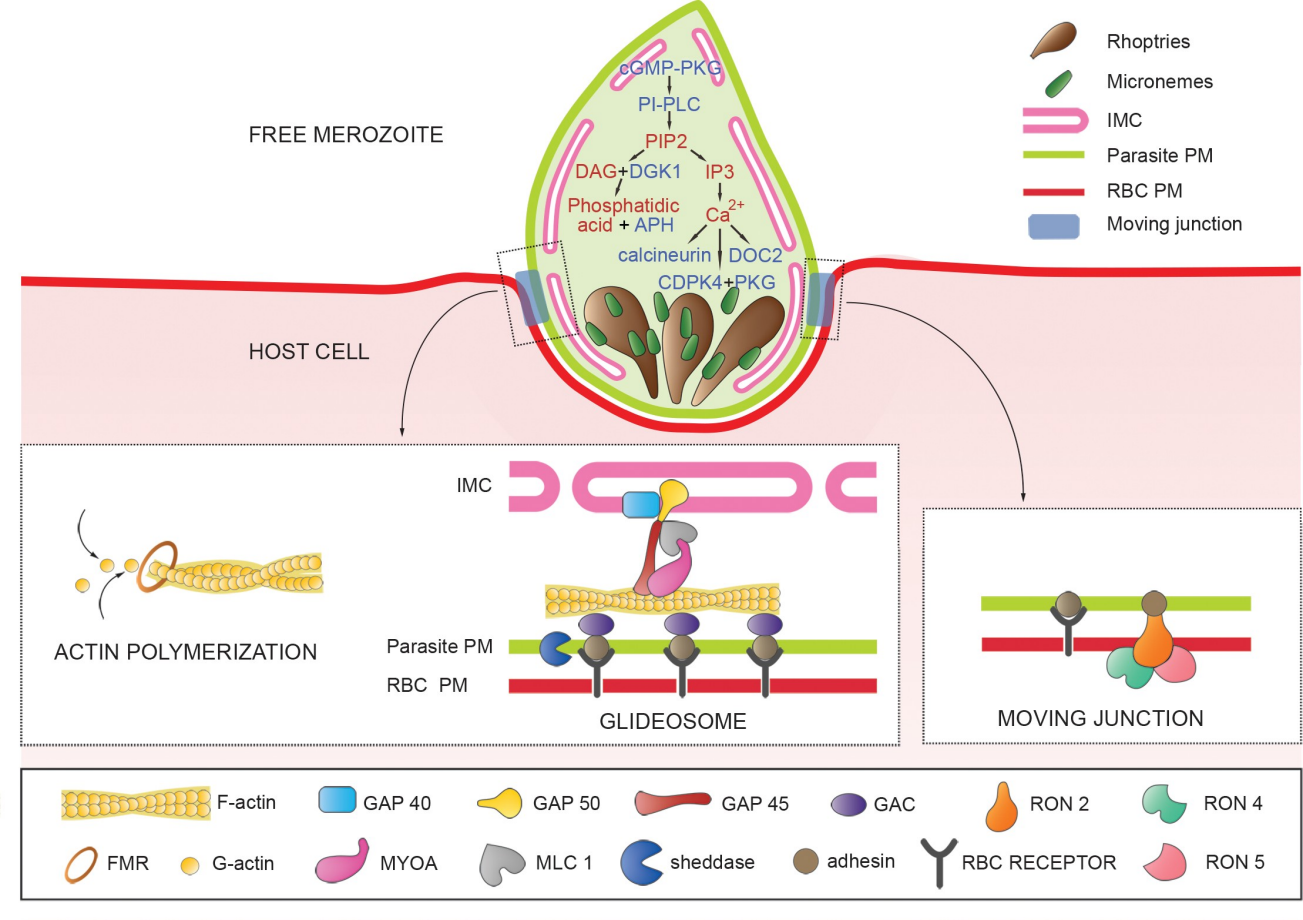
A molecular invasion model proposed for *B*. *divergens*. The scheme represents a hypothetical parasite-host complex that is translocated from the front of the parasite to the back by an actomyosin system during invasion. The free merozoite shows Ca^2+^–dependent proteins that could govern the secretion of transmembrane adhesive proteins from the micronemes during invasion in a Ca^2+^ dependent manner. These adhesins are translocated to the parasite plasma membrane (PM) and may act as ligands for host cell receptors. During invasion, parasite sheddases cleave adhesins to disengage interactions with RBC receptors. The scheme on the left shows a magnified view of the potential candidates related to the actin polymerization and glideosome, which localized between the inner membrane complex (IMC), the parasite PM and the membrane of the red blood cell (RBC-PM) powers the merozoite motile process. The scheme on the right shows a magnified view of the *B*. *divergens* potential candidates that facilitate parasite-RBC receptor connection and allow the moving junction formation. FMR, formin; GAP40, gliding associated protein 40; GAP45, gliding associated protein 45; GAP50, gliding associated protein 50; MYOA, myosin motor protein; MLC1, myosin light chain1; GAC glideosome-associated connector; RON2, rhoptry neck protein 2; RON4, rhoptry neck protein 4; RON5, rhoptry neck protein 5.

Since Ca^2+^-dependent proteins and Ca^2+^ signaling are important for microneme exocytosis and invasion in apicomplexan parasites [22], we identified the following *B*. *divergens* candidates genes that encode proteins related to Ca^2+^: i) BDIVROU_0339410.t1.2 (protein kinase G, PKG), ii) BDIVROU_0124800.t1.2 (phosphoinositide phospholipase C, PI-PLC), iii) BDIVROU_0071500.t1.1 (diacylglycerol kinase 1, DGK1), iv) BDIVROU_0291600.t1.1 (acylated pleckstrin homology domain containing protein, APH), v) BDIVROU_0224310.t1.2 (Ser/Thr phosphatase, PP1 or calcineurin), vi) BDIVROU_0130101.t1.2 (calcium-dependent protein kinase 4, CDPK-4) and vii) BDIVROU_0416400.t1.1 (double C2 domain protein, DOC2) (Table 2 and Fig 4). The identification of these genes suggests that Ca^2+^ and calcium–dependent proteins are involved in *B*. *divergens* invasion in a similar manner to other apicomplexans. Although PKG activates microneme secretion and parasite egress in a calcium-independent manner [25], this protein is associated with the release of Ca^2+^ from the endoplasmic reticulum (ER) to the cytoplasm and is mediated by cyclic guanosine monophosphate (cGMP). PI-PLC is then activated by cGMP-dependent PKG, thus leading to hydrolysis of phosphatidylinositol 4,5-bisphosphate (PIP2) to diacylglycerol (DAG) and inositol 1,4,5-trisphosphate (IP3), each of which plays a different role [31,32]. DAG is converted to phosphatidic acid by DGK1. Phosphatidic acid is a mediator of microneme secretion and is associated with APH, a surface microneme protein that acts as a phosphatidic acid–detecting protein. Depletion of APH leads to blockade of micronemes and, consequently, to inactivation of the motile machinery of the parasite or glideosome during invasion [33]. Moreover, IP3 stimulates an influx of Ca^2+^ from the ER surface to the cytoplasm [31,34]. Simultaneously, this influx of Ca^2+^ activates calcium-dependent proteins as calcineurin, DOC2 and CDPK4 which interacts with PKG, thus favoring attachment of the merozoite to the RBC, the secretion of several micronemal proteins [35] and the control of the actomyosin motor to ensure an efficient gliding motility for the parasite [36].

b) *B. divergens* genes related to actin dynamics, glideosome and moving junction

The actomyosin-based molecular motor or glideosome and the moving junction facilitate the entry of apicomplexan parasites into the host cell. These molecular systems are well characterized in *Plasmodium* and *Toxoplasma* [23,25] and we found the following six implicated genes from both within *B*. *divergens* genome: i) BDIVROU_0025700.t1.2 (actin); ii) BDIVROU_0303400.t1.1 (formin-a, FMRa); iii) BDIVROU_0036900.t1.1 (formin-b, FMRb); iv) BDIVROU_0096510.t1.2 (actin-depolymerizing factor, ADF or cofilin); v) BDIVROU_0279300.t1.2 (profilin); and vi) BDIVROU_0073701.t1.2 (F-actin–binding protein or coronin) (Table 2 and Fig 4). The identification of these genes suggests that the movement of *B*. *divergens* during invasion is mediated by actin in a similar manner to other apicomplexan parasites [37]. Actin is found as a globular monomer or G-actin and polymerizes into filaments of F-actin via a nucleation phase mediated by formins that localize at the apical pole of invading merozoites [38]. In contrast, ADF and profilin help to maintain a high actin content in the form of a monomer (G-actin) and a low concentration of polymerized actin (F-actin) by sequestering free G-actin [39–42] while coronin participates in the organization of F-actin filaments, the regulation of the motility [43] or mediating multiple parasites functions [44].

Actin dynamics and glideosome power the parasite locomotion [25], thus we identified six *B*. *divergens* genes related to the glideosome machinery: i) BDIVROU_0242901.t1.2 (myosin heavy chain A, MYOA); ii) BDIVROU_0412101.t1.2 (myosin light chain 1, MTIP for *Plasmodium* spp and MLC1 for *T*. *gondii*); iii) BDIVROU_0339200.t1.2 (gliding associated protein 50, GAP50); iv) BDIVROU_0272901.t1.2 (gliding associated protein 45, GAP45); v) BDIVROU_0052401.t1.2 (gliding associated protein 40, GAP40); and vi) BDIVROU_0152200.t1.2 (glideosome-associated connector, GAC) (Table 2 and Fig 4).

Similar to other apicomplexan parasites, the glideosome of *B*. *divergens* may be located between the plasma membrane and the inner membrane complex (IMC) of the parasite and comprises a similar molecular structure formed by MYOA, which in association with MLC1 [45,46], GAP45, GAP50 and GAP40 generates the traction force for motility and entry [38,47]. As an alternative to the MYOA motor, we predicted a second gene BDIVROU_0133800.t3.2 that encodes the myosin heavy chain B (MYOB), and it may play a similar role in parasite motility, thus suggesting that the movement in *B*. *divergens* could be governed by alternative myosin motors [48].

GAP proteins anchor the *B*. *divergens* glideosome to the IMC and the cytoskeleton [47,49,50], while the GAC connector attaches it to the plasma membrane [51]. This last protein specifically connects the glideosome to a transmembrane adhesin (TgMIC2 for *T*. *gondii*) that may interact with a host cell receptor to propel the parasite forwards [52]. In the *B*. *divergens* genome and transcriptomes, we found at least five adhesins as potential candidates to connect the parasite to the RBC: i) BDIVROU_0156200.t1.2 that encodes the orthologous thrombospondin-related apical membrane protein (TRAP for *Plasmodium* and TgMIC2 for *T*. *gondii*, mentioned above) [24]; ii) BDIVROU_0126000.t1.2 that codes for the apical membrane antigen BdAMA1, that binds to a trypsin- and chymotrypsin-sensitive receptor on the RBC [53]; iii) BDIVROU_0182900.t1.2 that encoded the glycosyl phosphatidyl inositol (GPI) anchored protein Bd37 involved in the penetration of *B*. *divergens* using a potential endocytosis mechanism [54,55]; iv) BDIVROU_0183000.t1.2 that codes for a putative 50-kDa surface protein (BdP50) and v) two identical copies (BDIVROU_0113100.t1.2 and BDIVROU_0112800.t1.1) of the genes that code the rhoptry-associated protein 1, RAP-1, a protein that binds to a RBC receptor [56].

In addition, we confirmed two more genes from the RAP family: i) BDIVROU_0112900.t1.2 that encoded a protein of 492 aa (RAP-1b) with an estimated molecular mass of 56.38 kDa, the amino acid sequence exhibited significant percentages of similarity to other known RAP proteins as *B*. *bovis* RAP-1 (83%) [57], *B*. *divergens* RAP-1 (72%) [56], *B*. *ovata* RAP-1 (79%) [58] and *B*. *gibsoni* RAP-1b (63%) [59] and ii) BDIVROU_0117600.t1.2 encoding a ropthry protein [60] that showed 34.4% similarity to the RAP-1 related antigen (RRA) of *B*. *bovis*, a protein that may contribute to erythrocyte invasion [61]. The amino acid sequence of RAP-1, RAP1-b and RRA from *B*. *divergens* exhibited the common features of the RAP family [61] such as a signal peptide, the presence of the strict conserved 4 cysteine residues and the 14 amino-acid motif. The genome sequence showed the *B*. *divergens rap-1* locus extending over about 7.3 Kb and contains two identical *rap-1* genes interspaced with the *rap-1b* gene. *Rap-1* and *rra* genes were located on the same contig but the *rra* gene was found around 96.8 kb downstream from the RAP-1 locus. None of the genes were flanked by other *rap-1* like genes or any gene related to the invasion or egress processes.

Moreover, for successful cell invasion, apicomplexan parasites establish a moving junction that moves from the apical to the posterior end of the parasite [23]. We identified at least three *B*. *divergens* genes implicated in the formation of this system: i) BDIVROU_0259100.t1. (rhoptry neck protein 2, RON2 [56]); ii) BDIVROU_0191501.t1.2 (rhoptry neck protein 4, RON 4); and iii) BDIVROU_0142300.t1.2 (rhoptry neck protein 5, RON5) (Fig 4).

Similar to *Plasmodium* and *Toxoplasma*, *B*. *divergens* RON proteins could constitute a ring-shaped structure in the host cell membrane. This structure together with AMA1 enables a tight connection between *P*. *falciparum* or *T*. *gondii* and their respective host cells leading to the formation of the moving junction [23,25,62–65]. However, as we mentioned above *B*. *divergens* AMA1 binds to a specific receptor on the RBC providing an attachment between the merozoite and the host cell [53], thus suggesting that the constitution of the moving junction core could be different between *B*. *divergens* and other species and could involve a host cell receptor. Alternative or multiple roles for *B*. *divergens* AMA1 and specific adaptation of this protein to a particular *B*. *divergens* merozoite-host cell interaction could be also considered [52].

Interestingly, we identified the gene BDIVROU_0165301.t1.2 (Table 2) that belongs to the spherical body protein family (SBP) of the genus *Babesia* which seem to be involved in stabilizing the environment after invasion and in aiding parasite growth [66]. We also identified the gene BDIVROU_0161200.t1.1 that encodes a subtilisin-like serine protease (BdSUB1) that play a relevant role during invasion [27] and five novel genes: i) BDIVROU_0278701.t1.2 (ROM4.1), ii) BDIVROU_0280300.t1.1 (ROM4.2), iii) BDIVROU_0439300.t1.1 (ROM4.3), iv) BDIVROU_0230400.t1.2 (ROM4.4) and v) BDIVROU_0449600.t1.1 (ROM4.5) that showed homology with rhomboid-like protease ROM4, suggesting the presence of a novel *B*. *divergens* ROM family.

The homologous *T*. *gondii* ROM4 and the *P*. *falciparum* subtilisin-like serine protease PfSUB2 cleaves the transmembrane domain of adhesins (AMA1, and TRAP) to disengage their interactions with the host cell for parasite entry [26,67,68]. Thus, the ROM protease family detected in the *B*. *divergens* asexual life cycle could aid in the shedding of surface adhesins as well.

In addition to shedding, *P*. *falciparum* AMA1 is phosphorylated by the cAMP-dependent protein kinase (cAMP-PKA) an enzyme that was recently shown to be involved in merozoite invasion [69]. This gene has also been identified for *B*. *divergens* in the present study (BDIVROU_0165500.t1.1).

c) *B. divergens* genes related to egress of the parasite

Egress is an ordered, regulated process assisted by a cascade of proteolytic activities that occur seconds before the parasite exits the host cell [24,70].

For example, perforins, that exhibit calcium-dependent lytic activity, form pores in the parasitophorous vacuole membrane of *Plasmodium* and in the plasma membrane of the host cell facilitating the egress process [28,29,71]. Interestingly, four *B*. *divergens* genes with homology to mac/perforin proteins (MAC) were also found in this work, these are: i) BDIVROU_0064900.t1.1 (MAC1), ii) BDIVROU_0161200.t1.1 (MAC2), iii) BDIVROU_0065210.t1.2 (MAC3), and iv) BDIVROU_0173601.t1.2 (MAC4) (Table 2). As reported in the literature, cysteine proteases are also involved in the asexual life cycle. Specifically, papain proteases play a dual role in haemoglobin digestion and parasite egress [72–74]. We identified eight *B*. *divergens* genes encoding cysteine proteases. Three of them belong to the papain family: i) BDIVROU_0308600.t1.1 (papain- 2), ii) BDIVROU_0202300.t2.1 (vignain like protease) and iii) BDIVROU_0197200.t1.1 (papain-like cysteine protease).

Interestingly, we also have curated the remaining 5 genes: i) DIVROU_0190900.t1.1 (calpain 7), ii), BDIVROU_0315500.t1.1 (CPC1-like protease), iii) BDIVROU_0028600.t1.1 (CPC2-like protease), iv) BDIVROU_0012300.t1.1 (*B*. *divergens* OTU‐like cysteine protease, BdOTU) and v) BDIVROU_0273700.t1.1 (*B*. *divergens* autophagy-related peptidase, BdATG4). These genes, have been previously described in *P*. *falciparum*, as essential for cell cycle progression [75] apicoplast homeostasis [76] and growth [77]. Therefore, the presence of these homologs suggests a similar role in *B*. *divergens*.

### Conclusions

Our study provides novel and comprehensive information on the identification of a large panel of proteins involved in the critical processes of invasion and egress of *B*. *divergens*. The strategy developed shows how genome sequencing and RNAseq can further our understanding of transcriptional patterns of *B*. *divergens* blood stages. Although apicomplexan genomes differ in terms of sequencing data and assembly strategy, orthologous proteins related to biological processes that have been reported for other parasites can be found in *B*. *divergens*.

Thus, we have proposed multiple invasion and egress molecular candidates for *B*. *divergens* guided by the cross-comparison with the related Apicomplexan parasites, *Plasmodium* and *Toxoplasma*. Based on our results, we confirmed significant similarities with both parasites but also some key differences in the composition of the *B*. *divergens* invasion machinery (Fig 4). Therefore, future studies are needed for defining conserved versus evolutionary adaptations of the principal molecules involved in these biological processes between Apicomplexan species. Moreover, our transcriptomic analysis revealed the expression profile of the *B*. *divergens* genome and showed which genes are active at a particular point in time. However, the genetic expression landscape observed in *B*. *divergens* free merozoite and intracellular life stages are pieces of a complex jigsaw puzzle that needs to be solved by using other techniques such as proteomics and metabolomics.

Furthermore, we will extend our analysis to the transcriptome of other clinical samples and/or isolates, especially those from hosts infected by *B*. *divergens*. Thus, we will be able to compare differences between the functional expression profile of the parasite *in vivo* during active infection and *in vitro*. Finally, we consider that the results generated in this work, can guide other efforts in the improvement of diagnosis and detection, drug target and vaccine development for the control of this emerging and neglected disease.

## Methods

### Ethics statement

Human A+ blood from healthy volunteer donors was used to maintain *B*. *divergens* blood stage cultures. Protocols for the use of this blood were approved by the Blood Transfusion Center, Madrid, Spain. Donors provided informed written consent for use of their blood for research purposes.

### *In vitro* culture of *B*. *divergens*

*B*. *divergens* (Bd Rouen 1987 strain) was propagated *in vitro* in human A+ RBCs. High parasitemia asynchronous parasite cultures were prepared at 40% parasitemia according to previously published protocols [9,78].

### Pulse field gel electrophoresis (PFGE)

PFGE was used to determine chromosome number and size of the *B*. *divergens* genome. Asynchronous cultures were centrifuged at 1224 x g for 5 min and washed with RPMI 1640 (Life Technologies Corporation, Carlsbad, CA). The resultant pellets, containing intact cells, were embedded in 0.8% agarose plugs using the clamped homogeneous electric field (CHEF) mammalian genomic DNA plug Kit and treated with proteinase K according to the manufacturer’s instructions (Bio-Rad Labs Inc., Hercules, CA). The CHEF-DR III system (Bio-Rad labs Inc.) was used to separate the intact chromosomes of *B*. *divergens*. The PFGE conditions used for a 0.1–2.0 Mb DNA size range were: 6 V cm-1, pulses of 60–120 s for 24 h and an angle of 120°. The conditions used for a 1.8–4.6 Mb DNA size range were: 4.5 V cm-1, pulses of 200 s for 48 h and an angle of 106°. The gels were stained and photographed under ultraviolet transillumination.

### Genome assembly, post-assembly improvement, gene prediction and annotation

The *de novo* genome assembly was performed using a hybrid approach with three different sequencing technologies. The first *de novo* assembly was obtained using the Newbler software [79] with 454 sequencing data and default parameters. Then, a second *de novo* assembly with Illumina paired-end and mate paired sequencing data, was performed using AllPaths-LG assembler [80] using default parameters. Then, 454 and Illumina scaffolds were merged using the genome assembler, reconciliation and merging (GARM) meta-assembler [81] with default parameters to generate a consensus assembly. Subsequently, SSPACE [82] and iterative mapping and assembly for gap elimination (IMAGE) [83] were used to improve scaffolding and gap filling, respectively. Finally, we used PacBio sequencing data that was previously corrected with Iterative Correction of Reference Nucleotides (iCORN) [84] using five iterations with Illumina reads, to increase the continuity of the assembly using the software Patch [80] with default parameters. The latest version of the genome can be found with the accession number GCA001077455 (CCSG02000001-CCSG02000141) and associated to the BioProject PRJEB6536.

For gene prediction, we used Braker v2.0.4 and AUGUSTUS v3.0.3 using a set of proteins that were obtained from the previous version of the assembly [15] and with the support of RNAseq data. We corroborated the predicted gene models by comparing them against proteins from other *Babesia* species deposited in the NCBI database and filtering them to 90% identity to remove redundancy. Using Proteinrtho v5.15 [85], we retained those models with at least one ortholog in other species. The following list of *Babesia* species were used: *B*. *bennetti*, *B*. *major*, *B*. *odocoilei*, *B*. *ovata*, *B*. *orientalis*, *B*. *capreoli*, *B*. *ovis*, *B*. *canis*, *B*. *rossi*, *B*. *rodhaini*, *B*. *equi*, *B*. *caballi*, *B*. *divergens*, *B*. *bigemina*, *B*. *gibsoni*, *B*. *microti and B*. *bovis*. Finally, protein annotation was achieved using the Trinotate v3.0.1 pipeline [86].

### Comparative genomics

For the comparative genomic analysis, the genomes available at Genbank for *B*. *bovis* (GCA_000165395.1) and *B*. *microti* (GCA_000691945.2) were used. The Proteinortho v5.15 [85] was used to obtain protein clusters of *Babesia* genomes analyzed here with the following parameters: E-value for blast: 1e-05, min. percent identity of best blast alignments: 25, min. coverage of best blast alignments in percent: 50, min. similarity for additional hits: 0.95. The results from the protein orthologous was also used to verify the annotation. Additionally, we used the Circos [87] package for circular data visualization and we were able to observe chromosome rearrangements among *B*. *divergens*, *B*. *bovis* and *B*. *microti*.

### Isolation of *B*. *divergens* parasites and RNA preparation

Free merozoites and intraerythrocytic parasites were collected from two highly parasitized independent asynchronous *B*. *divergens* cultures, 75 ml each at parasitemias of ≈ 40%. Cultures were centrifuged at 600 x g and 4°C for 5 min in fixed-angle rotor heads to collect the supernatant containing the free merozoites and pellets containing the intraerythrocytic forms. Free merozoites were isolated [9] and resuspended in Trizol LS Reagent (ThermoFisher). Pellets containing the intraerythrocytic forms were resuspended in RPMI 1640 and washed 3 times by centrifugation at 600 x g for 10 min in order to remove possible traces of free merozoites. Pellets were diluted to 1:3 with RPMI 1460. This suspension was then layered on top of a 1.122 g/ml isosmotic Percoll solution prepared according to the manufacturer´s instructions (GE Healthcare, Freiburg, Germany) and centrifuge (2000 x g, 10 min). The infected erythrocytes collected at the interface were washed 3 times in PBS at 4°C and 600 x g for 10 min and resuspended in Trizol LS Reagent (ThermoFisher). Total RNA from *B*. *divergens* free merozoites and intraerythrocytic parasites was prepared using Trizol LS Reagent (ThermoFisher) and chloroform extraction. DNA was removed using the QIAGEN RNase-Free DNase Set (Qiagen Inc. Valencia CA) and cytoplasmic ribosomal RNA was removed using the Ribo-Zero removal kit (Illumina, San Diego, CA, USA). The RNA was measured on an Agilent 2100 Bioanalyzer using an RNA 6000 Pico Chip kit (Agilent Technologies, Inc, Santa Clara, California) and used for libraries preparation and real-time PCR assays (S2 Fig.).

### RNA sequencing library preparation

Libraries were prepared using the Illumina TruSeq RNA Sample Prep Kit v2 (Illumina) following the manufacturer’s protocol. High quality RNA samples from free merozoites (FM1 and FM2) and from intraerythrocytic stages (IE1 and IE2), with RIN numbers ranged from 8.8 to 10, were used for NGS library construction (S2 Fig). FM1, FM2, IE1 and IE2 biological replicates were used to prepare two independent libraries for each stage. A third technical replicate was also prepared for each stage mixing FM1 and FM2 and IE1 and IE2 respectively. The libraries were sequenced using the Illumina HiSeq2000 platform with a paired-end configuration using 202 cycles (2x101 reads).

RNAseq data was deposited at NCBI under the Biosample accession number SAMN12187113 and included in the BioProject PRJNA552284.

### Differential expression analysis

Differential expression analysis was performed using two biological replicates through the integrated differential expression analysis multi-experiment (IDEAmex) website (http://zazil.ibt.unam.mx/ideamex/) using three different differential expression packages: edgeR [88], DESeq2 [89] and NOISeq [90]. EdgeR and NOISeq were performed by applying trimmed mean of M values (TMM) [91] as the normalization method. To identify differentially expressed genes, genes whose p value was less or equal to 0.01 and log fold change (FC) greater or equal than 1.5, were selected for each method. The genes reported as differentially expressed in Table 2, were inspected and curated manually using Artemis [92] and selected as candidates for further analysis.

### Real-time qRT-PCR verification of RNAseq data

Transcriptomic analyses were validated by real-time qRT-PCR using the same RNA extractions that had been prepared previously for RNAseq sequencing. Two μg each of free merozoite and intraerythrocytic parasite RNA was used for cDNA synthesis using the High Capacity RNA-to-cDNA kit (Applied Biosystems by ThermoFisher) following the manufacturer’s instructions. Controls without reverse transcriptase were used to investigate potential gDNA contamination by PCR using *actin-*specific primers. A set of 33 genes was tested by real-time qRT-PCR. Primers (S9 Table) were designed using Primer3 software [93] and were validated by testing amplification efficiency using 10-fold dilutions of cDNA by real-time qRT-PCR and melt curve analysis. Real-time qRT-PCR measurements were performed using the Qiagen Rotor-Gene-Q Real-Time PCR Detection System. Reactions were prepared in triplicated in a total volume of 20 μl using the SYBR Premix Ex Taq (Takara-Clontech, Otso, Japan), 20 ng of cDNA and primer concentrations of 0.2 mM. Transcript expression levels were calculated with the 2^−ΔC*t*^ method using the endogenous control gene BDIVROU_0242600.t1.1 encoding for *B*. *divergens* endonuclease/exonuclease/phosphatase family domain containing protein, EnExPh as references.

## Supporting information

S1 Fig Pulsed field gel electrophoresis (PFGE) of intact chromosomes using Clamped homogeneous electric field (CHEF) system. (A) The PFGE assay resolves the separation of 0.1–2.0 Mb DNA fragments and provides the approximate measurement of chromosome lengths in a 1% agarose gel: 1) *Saccharomyces cerevisiae* strain YNN295 DNA marker (the manufacture´s estimates of the sizes of *S*. *cerevisiae* chromosomes are indicated on the left of the picture); 2) *B*. *divergens* genomic DNA showing two bands at 1.05 Mb (chromosome 1) and 1.7–1.8 Mb (chromosome 2). (B) The PFGE assay resolves the separation of 1.8–4.6 Mb DNA fragments and provides the approximate measurement of chromosome lengths in a 0.8% agarose gel: 1) *Hansenula wingei* DNA marker; 2) *Schizosaccharomyces pombe* marker (the manufacture´s estimates of the sizes of *H*. *wingei* and *S*. *pombe* chromosomes are indicated on the left of the picture); 3) *B*. *divergens* genomic DNA showing two bands at 2.9–3.0 Mb (chromosome 3) and 4.0 Mb (chromosome 4).
(TIF)

S2 Fig Graphics show an analysis of *B*. *divergens* total RNA samples with the Agilent 2100 bioanalyzer, a principal component analysis (PCA) plot displaying variation and account of RNAseq samples and a multidimensional scaling (MDS) plot displaying the relative positions of RNAseq samples generated by DESeq2 and EdgeR, respectively. Panel A: The electrophoresis of *B*. *divergens* FM1, FM2, IE 1 and IE 2 RNA samples shows a visual inspection of RNA integrity. Panel B: RNA Integrity Number (RIN) of FM1, FM2, IE 1 and IE 2 RNA samples. Panel C: PCA plot of FM1, FM2, IE 1 and IE 2 RNAseq samples. Panel D: MDS plot of FM1, FM2, IE 1 and IE 2 RNAseq libraries. FM, free merozoite; IE, intraerythrocytic parasites.
(TIF)

S1 Table List of genes identified in *B*. *divergens* and their *B*. *bovis* and *B*. *microti* orthologous. Gene IDs for *B*. *bovis* and *B*. *microti* correspond to the NCBI annotation. #Species: 1, the gene was identified in one species; 2, the gene was identified in two species contain in their genomes; 3, the gene was identified in three species. Genes: total number of genes identified for the three species. Alg.Conn.: proteinortho algebraic connectivity. *absent gene.
(CSV)

S2 Table List of annotated *B*. *divergens* genes. The columns contain the BlastP, PFAM HMMER search, SignalP, KEGG, COG and EC number annotation results, respectively. For BlastP and PFAM results, the Evalue is attached with the “^” character, to the database target ID result. NA = not available.
(TXT)

S3 Table Genome sequences of the annotated genes from the *B*. *divergens* genome. (ZIP)

S4 Table RNAseq expression profile of both free merozoite and intraerythrocytic parasite stages using DESeq. (TXT)

S5 Table RNAseq expression profile of both free merozoite and intraerythrocytic parasite stages using EdgeR. (TXT)

S6 Table RNAseq expression profile of both free merozoite and intraerythrocytic parasite stages using NOISeq. (TXT)

S7 Table Nucleotide sequences in FASTA format (ffn). (FFN)

S8 Table Amino acid sequences in FASTA format (faa). (FAA)

S9 Table Verification of RNAseq results by real-time qRT-PCR. Sequence design of primers used in this study. For each gene, an 18–21 sense and antisense complementary oligonucleotide was generated.
(DOCX)
