# Supplementary figures and images for: Comparative and functional genomics of the protozoan parasite Babesia divergens highlighting the invasion and egress processes
Source: PLoS Negl Trop Dis. 2019 Aug 19;13(8):e0007680. doi: 10.1371/journal.pntd.0007680 (PMC6715253; doi:10.1371/journal.pntd.0007680)

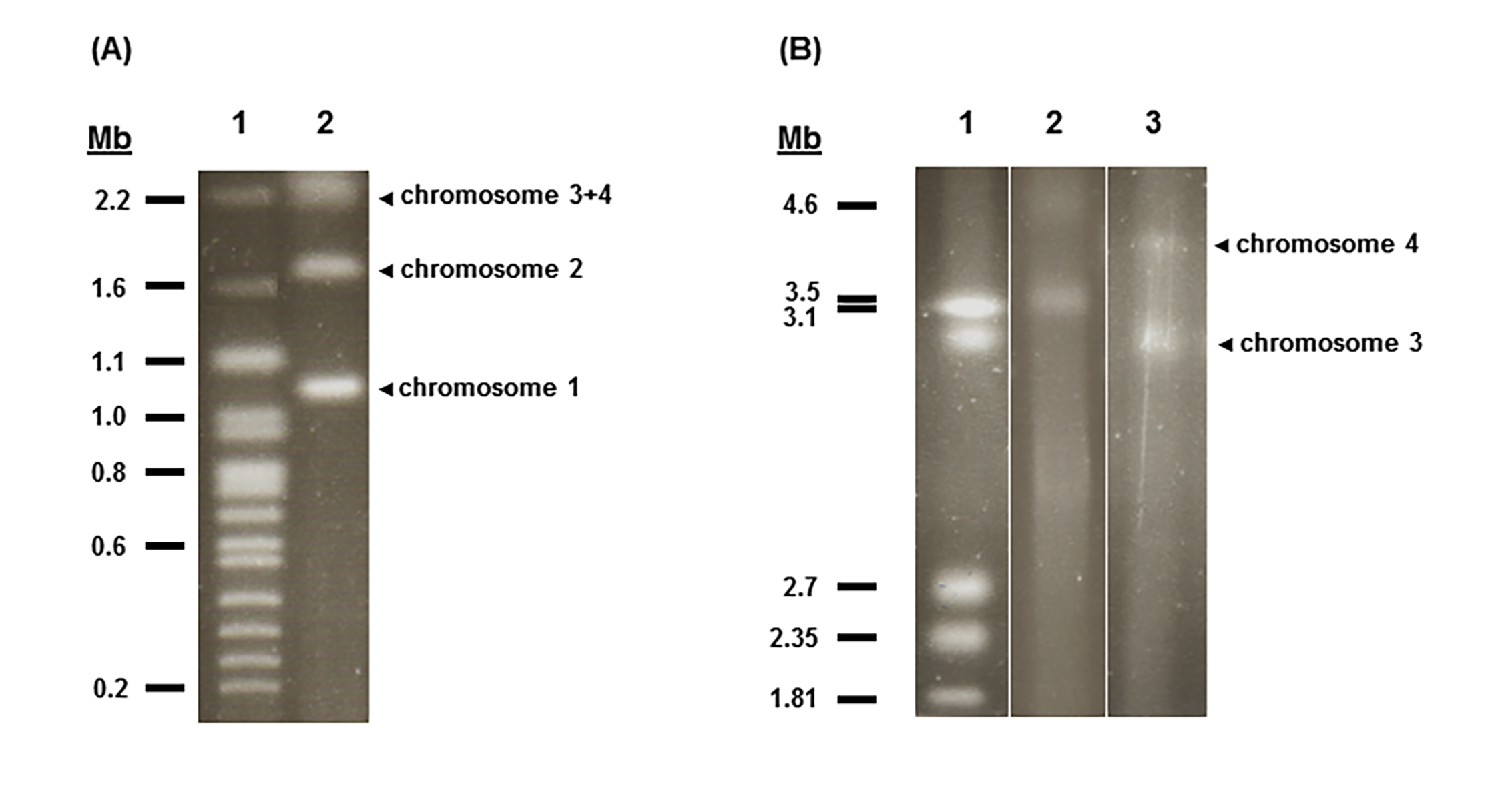

Supplement: S1 Fig — (A) The PFGE assay resolves the separation of 0.1–2.0 Mb DNA fragments and provides the approximate measurement of chromosome lengths in a 1% agarose gel: 1) Saccharomyces cerevisiae strain YNN295 DNA marker (the manufacture´s estimates of the sizes of S. cerevisiae chromosomes are indicated on the left of the picture); 2) B. divergens genomic DNA showing two bands at 1.05 Mb (chromosome 1) and 1.7–1.8 Mb (chromosome 2). (B) The PFGE assay resolves the separation of 1.8–4.6 Mb DNA fragments and provides the approximate measurement of chromosome lengths in a 0.8% agarose gel: 1) Hansenula wingei DNA marker; 2) Schizosaccharomyces pombe marker (the manufacture´s estimates of the sizes of H. wingei and S. pombe chromosomes are indicated on the left of the picture); 3) B. divergens genomic DNA showing two bands at 2.9–3.0 Mb (chromosome 3) and 4.0 Mb (chromosome 4). (TIF) [file pntd.0007680.s001.tif]

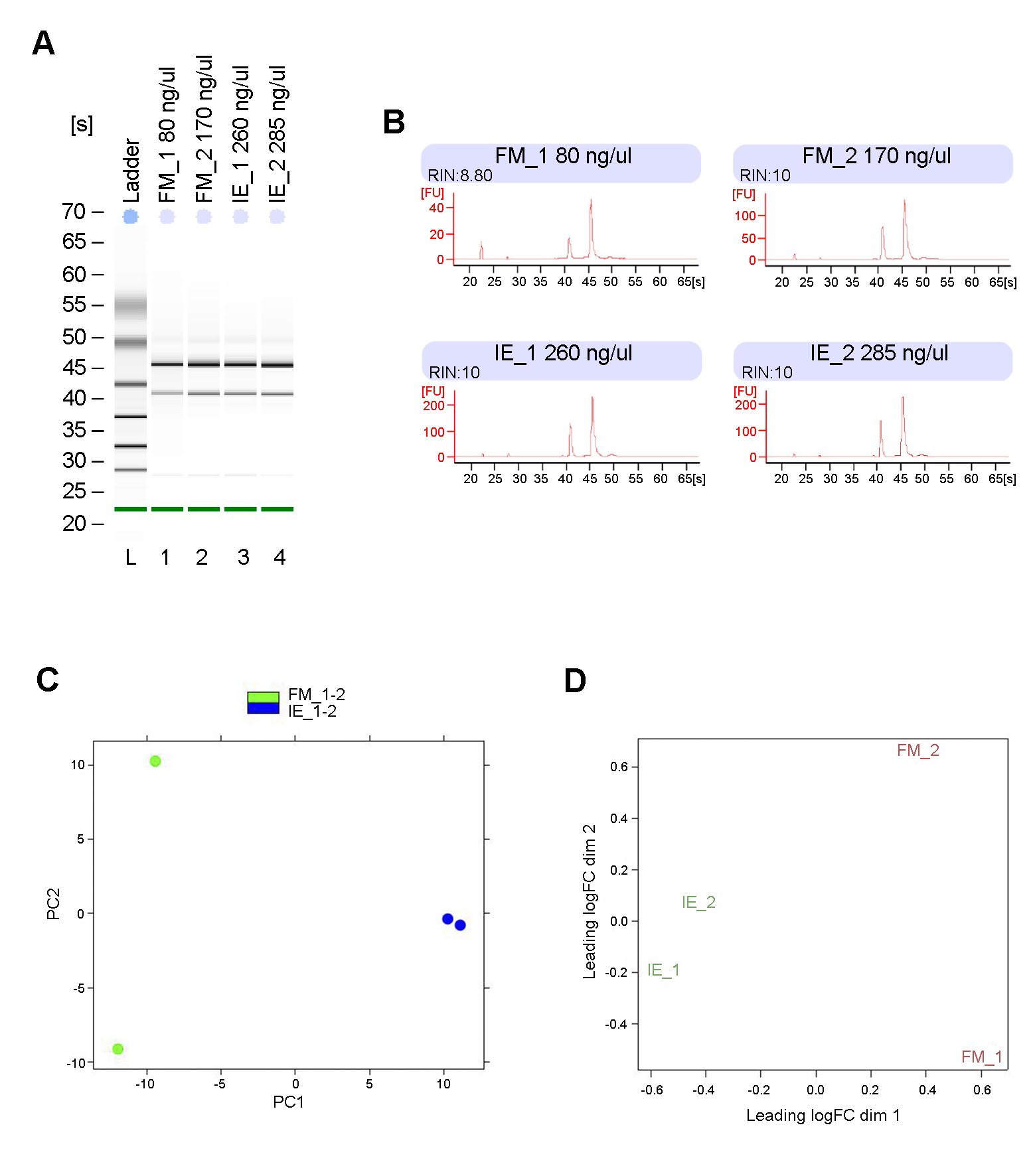

Supplement: S2 Fig — Panel A: The electrophoresis of B. divergens FM1, FM2, IE 1 and IE 2 RNA samples shows a visual inspection of RNA integrity. Panel B: RNA Integrity Number (RIN) of FM1, FM2, IE 1 and IE 2 RNA samples. Panel C: PCA plot of FM1, FM2, IE 1 and IE 2 RNAseq samples. Panel D: MDS plot of FM1, FM2, IE 1 and IE 2 RNAseq libraries. FM, free merozoite; IE, intraerythrocytic parasites. (TIF) [file pntd.0007680.s002.tif]
