# Supplementary material for: Comparative and functional genomics of the protozoan parasite Babesia divergens highlighting the invasion and egress processes
Source: PLoS Negl Trop Dis. 2019 Aug 19;13(8):e0007680. doi: 10.1371/journal.pntd.0007680 (PMC6715253; doi:10.1371/journal.pntd.0007680)
Supplement: S9 Table — Sequence design of primers used in this study. For each gene, an 18–21 sense and antisense complementary oligonucleotide was generated. (DOCX) [file pntd.0007680.s011.docx]

| **Gene ID** | **Gene** | **Forward Primer** | **Reverse Primer** |
| --- | --- | --- | --- |
| BDIVROU_0183000.t1.2 | 50-kDa surface protein, BdP50 | TACTCTGCTTGGTGAGGAGGC | TCGTGACATCAGTTCCAGCGG |
| BDIVROU_0182900.t1.2 | 37-kDa glycosylphosphatidylinositol-anchored surface protein, Bd37 | TTACCGTGCCGGAATACGGTG | AGGTACAGCAGCGAAGGATGG |
| BDIVROU_0126000.t1.2 | apical membrane antigen 1, BdAMA1 | AGCAGTTGGATCGCCTCTC | CAGAAGGCCCAGGATGAAG |
| BDIVROU_0272901.t1.2 | gliding associated protein 45, GAP45 | AGGAACAGCAGCCGTCAC | GAGGAGCGTCCGACAAATC |
| BDIVROU_0280000.t1.2 | subtilisin-like serine protease, BdSUB1 | CGTCGTACCCGCCATGTA | CACCCCCTCAGCAATAACC |
| BDIVROU_0165301.t1.2 | spherical body protein 3, SBP3 | GGGCTCCGGTGATAATGTG | CACGGTATTGACCCCTCCT |
| BDIVROU_0278701.t1.2 | rhomboid-like protease 4.1, ROM4.1 | TCTCTGTGGGGCCCTTATACC | CTGTCTCCTTTCGTCGCTCAG |
| BDIVROU_0230400.t1.2 | rhomboid-like protease 4.4, ROM4.4 | GATTCACGGCGCCACTACTAC | CACCAGTATCGAGCTCTCTGC |
| BDIVROU_0259100.t1.2 | rhoptry neck protein 2, RON2 | CGCTCTCTGTGACGGTGTT | GTGAGGGTTCCTGGGTCTTC |
| BDIVROU_0191501.t1.2 | rhoptry neck protein 4, RON4 | GGAGTTCCTGCATGTGGTC | CCTCTCTGCCGTTTTCGTG |
| BDIVROU_0142300.t1.2 | rhoptry neck protein 5, RON5 | GTCACCGTCAGAGGATACTGG | TATACGAGCCTGCCGTCAC |
| BDIVROU_0156200.t1.2 | thrombospondin-related apical membrane protein, TRAP | GAGAGAGAGTATGGCCACTCG | GTAGCTCTCGGCAGACTCGT |
| BDIVROU_0113100.t1.2 | rhoptry-associated protein 1, RAP-1 | GCAAGCGTGCTCCATCTTC | AGGTTCTCTCCGCTGCTCTC |
| BDIVROU_0112901.t1.2 | rhoptry-associated protein 1b, RAP1b | GTGCCGCTAGGTATCTTGGTC | GGTTGCTATCCCCCTGTTC |
| BDIVROU_0161201.t1.2 | mac/perforin protein 2, MAC2 | AGGCAGAGAGCCTTGCAC | GACCGTTTAGCCACAGACG |
| BDIVROU_0025700.t1.2 | actin | GGTATCGTGCTCGACTCCGG | TCCTCCTCGAAGTCGAGGGC |
| BDIVROU_0065210.t1.2 | mac/perforin protein 3, MAC3 | GCCCTGCTCACCAGAAGA | AGGGTGTAACCGGAGTTGC |
| BDIVROU_0173601.t1.2 | mac/perforin protein 4, MAC4 | CAGAGGTCTACGGCATCTCC | AGGAACAGTCGCCACACAC |
| BDIVROU_0052401.t1.2 | gliding associated protein 40, GAP40 | ATGTGGGAGAACATGGCAAG | AAGAAGCGACCAACGATGAC |
| BDIVROU_0412101.t1.2 | myosin light chain1, MLC1 | GCCACCGCATCTAACTTTGT | AGTGCTTGTACGCAATCGTG |
| BDIVROU_0073701.t1.2 | F-actin–binding protein or coronin | AACATCAAATACCGCCCATC | CTCCTTGTCAGCATCACACC |
| BDIVROU_0133800.t3.2 | myosin chain B, MYOB | TGCGACAATAAAGGGGTTTC | GTATGGGCAAAAGGCAGCTA |
| BDIVROU_0130101.t1.2 | calcium-dependent protein kinase 4, CDPK-4 | CGTGATCTCAAACCGGAGAA | CGGGAAGCCACAGAGTAAAA |
| BDIVROU_0124800.t1.2 | phosphoinositide phospholipase C, PI-PLC | CACCTAATGAAGGGCTGGAA | TTGCGGATTTTTCGATGACT |
| BDIVROU_0096510.t1.2 | actin-depolymerizing factor, ADF or cofilin | GGAAGTGGCGATGTTGATG | CTCCTCCACGAGGTGTTTGT |
| BDIVROU_0339200.t1.2 | gliding associated protein, GAP50 | GGCGTATCTAACTGGGCAAA | CTTTGTAGGGGAAAGCGTCA |
| BDIVROU_0339410.t1.2 | protein kinase G, PKG | TTATGTCAAGGACCAGGATGC | TTGCGATGAGAGAACCCTTT |
| BDIVROU_0242901.t1.2 | myosin A, MYOA | CGTTGTAAAGGCACTGTTCG | GGGCTGCTTTGAGTCGTTAG |
| BDIVROU_0224310.t1.2 | Ser/Thr phosphatase, PP1 or calcineurin | TCAGCAAAGCAGAACAGGAA | GCCATTGTGAGCAGTATCGTAA |
| BDIVROU_0279300.t1.2 | profilin | TTCTAGCATCGGCATCTTCA | GTATTCAGCAAGGGCAAAGG |
| BDIVROU_0152200.t1.2 | glideosome-associated connector, GAC | ATCCGCTGCATTGAGTCTTT | ATGTTGCCCTCCTTGTTGTC |
| BDIVROU_0117600.t1.2 | RAP-1 related antigen, RRA | TCAACGAAATCACGCTGTCT | GGTGTTCATGCCAGTTACCC |
| BDIVROU_0308600.t1.1 | papain-2 | AGGGGTACGACGCTGAAAC | CCTTTGCTCTGTGTGCTTTG |
| BDIVROU_0242600.t1.1 | endonuclease/exonuclease/phosphatase family domain containing protein, EnExPh | GGGCGAGGGTGATTTAGAG | CCGCAGAACCACGTAACC |
